# Effects of Antiviral Therapy on HBV Reactivation and Survival in Hepatocellular Carcinoma Patients Undergoing Hepatic Artery Infusion Chemotherapy

**DOI:** 10.3389/fonc.2020.582504

**Published:** 2021-02-01

**Authors:** Shousheng Liu, Jinfa Lai, Ning Lyu, Qiankun Xie, Huijiao Cao, Dabiao Chen, Meng He, Bei Zhang, Ming Zhao

**Affiliations:** ^1^ State Key Laboratory of Oncology in South China, Collaborative Innovation Centre for Cancer Medicine, Sun Yat-sen University Cancer Center, Guangzhou, China; ^2^ Department of the General Medicine, Sun Yat-sen University Cancer Center, Guangzhou, China; ^3^ Department of Minimally Invasive Interventional Radiology, Sun Yat-sen University Cancer Center, Guangzhou, China; ^4^ Department of Radiation Oncology, Nanfang Hospital, Southern Medical University, Guangzhou, China; ^5^ Department of Infectious Diseases, The Third Affiliated Hospital of Sun Yat-sen University, Guangzhou, China

**Keywords:** antiviral prophylaxis, hepatitis B virus reactivation, hepatic artery infusion chemotherapy, hepatocellular carcinoma, overall survival

## Abstract

**Background:**

This study aimed to investigate the influence of hepatic artery infusion chemotherapy (HAIC) on hepatitis B virus (HBV) reactivation in hepatitis B surface antigen (HBsAg) positive patients with primary hepatocellular carcinoma (HCC) as well as evaluate the role of antiviral prophylaxis in these patients.

**Methods:**

We enrolled 170 HBsAg-positive advanced HCC patients receiving HAIC using mFOLFOX regimen, of which 137 patients received antiviral prophylaxis. Risk factors for HBV reactivation were analyzed. The overall survival (OS) from the first application of HAIC were compared between antiviral and non-antiviral groups.

**Results:**

A total of 25 patients (14.7%) developed HBV reactivation after HAIC, of which 16 patients received antiviral treatment and nine patients did not. The incidence of HBV reactivation was 11.7% (16/137) in antiviral group and 27.3% (9/33) in non-antiviral group respectively. No antiviral prophylactic was the only significant risk factor for HBV reactivation (OR=12.35, 95% confidence interval (CI) 4.35–33.33, p<0.001). Patients in antiviral group received more cycles of HAIC compared with non-antiviral group (3.11 ± 1.69 vs 1.75 ± 1.18, p<0.05) at the time of HBV reactivated. Seven of the 25 HBV reactivation patients developed hepatitis. OS in antiviral group was significantly longer than that of non-antiviral group (median 16.46 vs 10.68 months; HR=0.57; 95% CI, 0.36–0.91; p<0.05).

**Conclusions:**

HBV reactivation is more prone to occur in the HBsAg-positive HCC patients undergoing HAIC without antiviral prophylaxis. Regular monitoring of HBV DNA and antiviral prophylaxis are suggested to prevent HBV reactivation as well as prolong the OS of these patients.

**Name of the Trial Register:**

HAIC Using Oxaliplatin Plus Fluorouracil/Leucovorin for Patients with Locally Advanced HCC.

**Clinical Trial Registration:**

https://www.clinicaltrials.gov/, identifier NCT 02436044

## Introduction

Hepatocellular carcinoma (HCC) is the fifth most frequently diagnosed cancer and the third most common cause of cancer-related death (1). Among all the etiological factors, chronic hepatitis B virus (HBV) infection is a major risk factor for HCC in Asia and sub-Saharan Africa patients. The risk of developing HCC in HBV carriers is 100 times greater than those who have not been infected with HBV (2). According to guideline of Chinese Medical Association of Infectious Diseases (3), antiviral treatment is recommended for hepatitis B surface antigen (HBsAg) positive patients with detectable HBV DNA if their serum alanine transaminase (ALT) level consistently exceeds the normal upper limit without other causes. At present, there is no unified guideline for the use of antiviral treatment in HBV-related HCC (4), and the clinical practice of antiviral therapy for HBV-related HCC mainly refers to the management of chronic hepatitis B (CHB).

At present, there is no absolutely uniform diagnostic standard for HBV reactivation at home and abroad, basically a consensus has been reached: HBV reactivation is characterized by the reappearance of HBV DNA or a 10-fold or greater increase in HBV DNA level compared to the baseline level, and it rarely happened in CHB patients or asymptomatic HBV carriers until some interventions such as local or systemic treatment break the balance between the virus and organism (5, 6). HBV reactivation may be asymptomatic or accompanied by elevated serum ALT levels and active hepatitis, thus may result in the slowdown or interruption of treatment, and then delaying the valid treatment of cancer patients that would exert great influence on the prognosis. Occasionally, it may cause lethal hepatic failure (7, 8).

It has been reported that HBV reactivation can occur in HCC patients underwent treatment such as systemic chemotherapy, immunotherapy, radiotherapy, transarterial chemoembolization (TACE), hepatectomy and radiofrequency ablation (RFA), which can be effectively prevented by antiviral therapy (9–15). Therefore, although no uniform global guidelines for the use of antiviral therapy at present, antiviral therapy is recommended for HCC patients receiving above locoregional or systemic treatment. Hepatic artery infusion chemotherapy (HAIC), especially in Asia, has been applied to advanced stage HCC in order to improve the therapeutic efficacy. It has been reported that HAIC using mFOLFOX regimen yielded significantly better therapeutic response than did TACE and sorafenib, and might represent a promising first-line treatment for HCC patients in advanced stage (16, 17). Besides, HAIC plus sorafenib further improved the overall survival compared with single sorafenib in HCC patients with portal vein invasion (18). HAIC allows for continuous and direct infusion of chemotherapeutic drugs into the tumor feeding vessels, thus increasing the local drug concentration and minimizing systemic toxicities through the strong first-pass effect in the liver (19, 20). The complications of HAIC were reported as jaundice, fever, gastrointestinal symptoms (nausea, vomiting and abdominal pain), infection, thrombosis and so on (21). However, the prevalence of HBV reactivation after HAIC and whether antiviral prophylaxis can prevent HBV reactivation remains unclear.

In the present study, we enrolled 170 HBsAg positive HCC patients who underwent HAIC using mFOLFOX to determine the incidence and risk factors of HBV reactivation after HAIC. We also assessed the effect of antiviral prophylaxis on HBV reactivation and survival in these patients.

## Patients and Methods

### Patients

**Figure 1:**
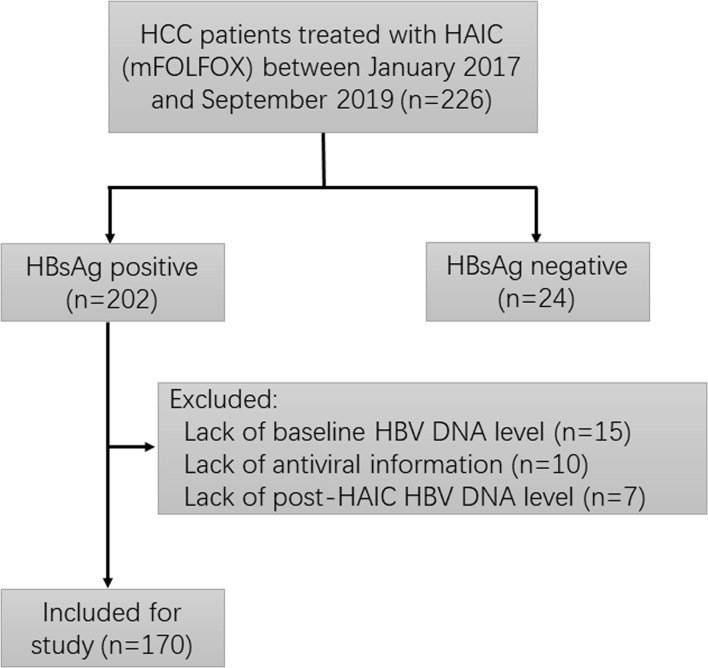
Flowchart depicting patient deposition. HCC, hepatocellular carcinoma; HAIC, hepatic artery infusion chemotherapy; HBsAg, hepatitis B surface antigen; HBV, hepatitis B virus.

As part of an ongoing phase II clinical trial (NCT 02436044) assessing the safety and efficacy of HAIC using mFOLFOX (oxaliplatin plus fluorouracil/leucovorin) for patients with locally advanced HCC, 226 patients diagnosed with advanced primary HCC (BCLC-C stage) and received HAIC using mFOLFOX regimen at Sun Yat-sen University Cancer Center were screened for eligibility in this prospective investigation. The inclusion criteria include (1): diagnosed with advanced HCC; (2) received at least one cycle of HAIC (mFOLFOX) therapy; (3) positive for HBsAg; and (4) a more than 3 months follow-up period after HAIC. Patients were excluded with any of the following evidences: accompanied with other hepatotropic viruses or human immunodeficiency virus (HIV); lack of baseline or post-HAIC HBV DNA levels; and lack of antiviral information. Among these patients, 56 patients were excluded for the following reasons: HBsAg negative (n=24), lack of baseline HBV DNA level (n=15), lack of antiviral information (n=10), and lack of post-HAIC HBV DNA level (n=7). The remaining 170 patients were finally recruited into this study. The screening process of enrolled patients is summarized in **Figure 1**.

Clinical indicators of enrolled patients before HAIC were evaluated as follows: age, gender, tumor number and size, Child–Pugh classification, serum alpha-fetoprotein (AFP), ALT, serum HBV DNA level, and hepatitis B serology. Follow-up laboratory examination indexes, including liver function test, HBV serology and HBV DNA quantification were performed every 3 weeks during HAIC and every 4 to 8 weeks for 3 months after the completion of therapy. Hepatitis B serology markers were detected by commercial enzyme immunoassays (Auszyme MC Dynamic, North Chicago, USA). Serum hepatitis B loads were measured using HBV DNA quantitative kit (DaAn Gene, Guangzhou, China) with a lower detection limit of 0 IU/ml.

### HAIC Procedure

All patients received a three-week cycle regimen. The HAIC protocol was as follows: A catheter was inserted into the hepatic artery followed by a microcatheter selectively placed into the feeding arteries of the tumor. Then, the microcatheter was connected to the arterial perfusion pump and the following treatment (mFOLFOX) was performed: oxaliplatin 130 mg/m^2^, leucovorin 400 mg/m^2^ and 5-FU 400 mg/m^2^ (10 min after leucovorin) intra-arterial infusion respectively on day 1, followed by 5-FU 2,400 mg/m^2^ continuous arterial infusion for 46 h. After HAIC completed, the indwelling catheter and sheath were removed, and pressure bandage was performed to stanch bleeding.

HAIC was repeated until tumor stable or progression, or intolerable toxicity was observed, or until the patients declined to continue the treatment.

### Definitions

HBV reactivation was defined as a 10-fold or more increase in serum HBV DNA level compared with baseline or reappearance of HBV DNA from an undetectable level (0 IU/ml) at baseline and a post-treatment HBV DNA > 200 IU/ml (22).The definition of hepatitis is that the level of serum ALT is three times or more higher than the upper limit of normal value (< 40 IU/L), or the absolute value of ALT increases above 100 IU/L. Hepatitis was attributed to HBV reactivation when there was evidence that hepatitis accompanied with HBV reactivation, in the absence of co-infection with other hepatitis viruses or other systemic infections, as well as exclusion of drug-induced liver injury (23, 24). Antiviral prophylaxis was defined as anti-HBV treatment (nucleoside analogs, NAs) administered before and during HAIC. Overall survival (OS) referred to the time from first application of HAIC to death due to HCC.

### Statistical Analysis

Statistical analyses were performed using SPSS 22.0 software (SPSS Inc, USA). Categorical characteristics between antiviral and non-antiviral groups were compared using the Pearson Chi square test. Two categories logistic regression model was used to predict the odds ratio (OR) of risk factors for HBV reactivation. Only variables with p value of less than 0.1 in the univariate model were included for further analysis in the multivariate model. Survival probabilities was estimated using the Kaplan-Meier method and survival curves were compared between groups by log-rank test. A p value of less than 0.05 was considered statistically significant.

## Results

### Patient Characteristics

A total of 170 HBsAg positive HCC patients were enrolled in the study, including 137 patients receiving antiviral prophylaxis and 33 patients receiving no antiviral prophylaxis. Prophylactic antiviral treatment continued until the patients died, with a median duration of 18.23 months. The clinical baseline features of the 170 patients based on antiviral prophylaxis or not are shown in **Table 1**. The majority of the patients were male (n=136, 91.8%) and the median age was 51 years (range, 28–75 years). Patients with liver function of Child-Pugh class A (n=156, 91.8%) accounted for the majority, while no patients had Child-Pugh class C liver function. The median number of HAIC cycles received was 2.0 (range, 1–10 cycles). At baseline, 126 patients (74.1%) had detectable HBV DNA level, among whom 86.5% (n=109) received antiviral prophylaxis; while among other 44 patients with undetectable HBV DNA level, only 63.6% (n=28) took antiviral prophylaxis. The median value of serum HBV DNA in the patients with detectable levels was 2.93×10^3^ IU/ml. Details of antiviral drugs used in enrolled patients are shown in **Table 2**. Of the four antiviral drugs, entecavir was the most frequently used agent (n=119, 86.9%). All factors were balanced between the two groups in statistics at baseline except for HBV DNA level (p=0.001) and HBeAg expression (p=0.049).

**Table 1:** Baseline characteristics of hepatocellular carcinoma (HCC) patients based on antiviral prophylaxis used or not.

Characteristics	Non-antiviral group(N=33)	Antiviral group(N=137)	p-value
Number of cases (n, %)			
Age (years)			0.069
≤51	12 (36.4)	74 (54.0)	
>51	21 (63.6)	63 (46.0)	
Gender			0.613
Male	31 (93.9)	125 (91.2)	
Female	2 (6.1)	12 (8.8)	
Tumor number			0.356
1-3	9 (27.3)	49 (35.8)	
>3	24 (72.7)	88 (64.2)	
Tumor size (cm)			0.357
≤5	7 (21.2)	40 (29.2)	
>5	26 (78.8)	97 (70.8)	
Child-Pugh class			0.613
A	31 (93.9)	125 (91.2)	
B	2 (6.1)	12 (8.8)	
AFP (ng/ml)			0.738
<400	16 (48.5)	62 (45.3)	
≥400	17 (51.5)	75 (54.7)	
HAIC (cycles)			0.092
≤2	22 (66.7)	69 (50.4)	
>2	11 (33.3)	68 (49.6)	
ALT (U/L)			0.381
≤40	16 (48.5)	78 (56.9)	
>40	17 (51.5)	59 (43.1)	
HBV DNA level			**0.001**
Undetectable	16 (48.5)	28 (20.4)	
Detectable	17 (51.5)	109 (79.6)	
HBeAg			**0.049**
Negative	30 (90.9)	103 (75.2)	
Positive	3 (9.1)	34 (24.8)	

The bold type indicates that the P value is statistically significant.

**Table 2:** Details and outcome of the enrolled patients based on antiviral agents.

Antiviral agents	No. of patients (%)	No. of HBV reactivation events
Entecavir	119 (70.0%)	8
Lamivudine	4 (2.4%)	1
Telbivudine	6 (3.5%)	0
Adefovir	8 (4.7%)	0
Non	33 (19.4%)	16

Non, no antiviral agents used.

### Risk Factor Analysis for HBV Reactivation

We conducted the logistic regression analysis to explore the risk factors for HBV reactivation. As shown in **Table 3**, AFP less than 400, undetectable HBV DNA level and no prophylactic antiviral treatment were risk factors for HBV reactivation in univariate model (all p<0.1). However, when it came to multivariate model, no antiviral prophylactic was the only significant risk factor for HBV reactivation (OR=12.35, 95% confidence interval (CI) 4.35–33.33, p<0.001).

**Table 3:** Logistic regression analysis of risk factors for hepatitis B virus (HBV) reactivation.

Variables	Univariate analysis		Multivariate analysis	
	OR (95% CI)	p-value	OR (95% CI)	p-value
Age (> 51)	0.78 (0.33–1.82)	0.559		
Gender (Female)	0.96 (0.20–4.59)	0.963		
Tumor number (> 3)	1.40 (0.55–3.56)	0.486		
Tumor size (> 5cm)	0.78 (0.31–1.96)	0.599		
Child-Pugh class (B)	0.42 (0.05–3.39)	0.418		
AFP (≥ 400ng/mL)	0.42 (0.17–1.01)	**0.054**	0.36 (0.13–1.01)	0.052
HAIC (> 2cycles)	0.73 (0.31–1.74)	0.484		
ALT (> 40U/L)	0.66 (0.27–1.58)	0.345		
HBV DNA level (Detectable)	0.25 (0.10–0.61)	**0.002**	0.41 (0.15–1.12)	0.082
HBeAg (Positive)	1.16 (0.43–3.16)	0.769		
Antiviral prophylaxis (Yes)	0.075 (0.03–0.20)	**0.000**	0.081 (0.03–0.23)	**0.000**

OR, odds ratio; 95% CI, 95% confidence interval.

Only the meaningful factors (p < 0.1) in univariate analysis were brought into the multivariate analysis. The bold type indicates that the P value is statistically significant.

### Clinical Features of Patients in HBV Reactivation Subgroup

We further analyzed the clinical characteristics of patients with HBV reactivation based on whether they received antiviral prophylaxis or not. A total of 25 patients (14.7%) developed HBV reactivation after HAIC, of which 16 patients received antiviral prophylaxis and nine patients did not. The incidence of HBV reactivation was 11.7% (16/137) in antiviral group and 27.3% (9/33) in non-antiviral group respectively. The baseline characteristics are summarized in **Table 4**. No obvious differences were observed between the two groups, except that patients in antiviral group received more cycles of HAIC compared with non-antiviral group (3.11 ± 1.69 vs 1.75 ± 1.18, p<0.05) when HBV reactivated.

**Table 4:** Baseline characteristics of hepatocellular carcinoma (HCC) patients based on antiviral prophylaxis used or not in the hepatitis B virus (HBV) reactivation subgroup.

Characteristics	Non-antiviral group (n = 16)	Antiviral group (n = 9)	p-value
Age (years)	50.25 ± 14.11	48.00 ± 11.18	0.685
Gender (M/F)	16/0	7/2	**0.049**
Tumor number (≤3/> 3)	5/11	2/7	0.629
Tumor size (cm)	8.20 ± 4.43	5.41 ± 2.70	0.101
Child-Pugh class (A/B)	16/0	8/1	0.174
AFP (< 400/≥ 400)	10/6	6/3	0.835
HAIC (cycles)	1.75 ± 1.18	3.11 ± 1.69	**0.027**
ALT (U/L)	39.98 ± 21.58	31.90 ± 12.60	0.317
HBV DNA (undetectable/detectable)	7/9	6/3	0.271
HBeAg (negative/positive)	14/2	5/4	0.073

The bold type indicates that the P value is statistically significant.

### Details of Patients With Hepatitis Attributed to HBV Reactivation After HAIC

Seven of the 25 HBV reactivation patients developed hepatitis, which also means HBV related hepatitis. The details of the seven patients are listed in **Table 5**. All of them were male, with a median age of 59 years (range, 32–71) and four of them received no antiviral agents. The median cycles of HAIC received was two (range, 1–5 cycles). The baseline HBV DNA level was mostly undetectable (median 0 IU/ml, ranging from 0 to 1.9×10^3^ IU/ml). ALT level increased three times or more compared with baseline level, while no significant alteration in total bilirubin was found. The incidence of hepatitis due to HBV reactivation was significantly higher in the non-antiviral group than that in the antiviral group (12.1% [4/33] vs 2.3% [3/133], p<0.05), whereas the incidence of hepatitis in HBV reactivation subgroup was comparable between antiviral and non-antiviral groups (33.3%[3/9] vs 25%[4/16], p=0.656). None of the patients died due to hepatitis, and most of the patients’ liver function recovered to relatively normal level after antiviral and supportive treatment.

**Table 5:** Clinical features of seven patients with hepatitis attributed to hepatitis B virus (HBV) reactivation after hepatic artery infusion chemotherapy (HAIC).

Patient	Gender	Age (years)	HAIC cycles	Antiviral agents	Baseline			At diagnosis of HBV reactivation		
					HBV DNA (IU/ml)	ALT (U/L)	Total bilirubin (umol/L)	HBV DNA (IU/ml)	ALT (U/L)	Total bilirubin (umol/L)
1	Male	59	2	Lamivudine	0	17.1	10.5	2,310	133.2	9.9
2	Male	42	2	Entecavir	0	22.6	9.3	4,980	224.6	14.7
3	Male	66	3	Entecavir	0	23.9	13.9	3,010	136.3	9.8
4	Male	39	1	Non	0	31.6	34.7	5,080	157.9	29.9
5	Male	64	3	Non	1,900	25.0	12.8	26,000	236.0	10.4
6	Male	32	5	Non	4	20.0	16.5	2,850	167.5	7.3
7	Male	71	2	Non	10	15.2	6.7	40,800	153.1	5.3

### Survival Analysis

**Figure 2:**
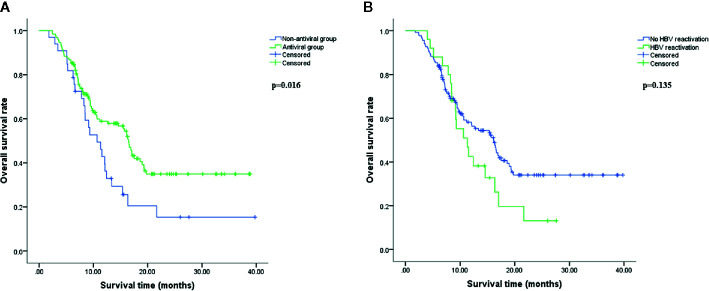
Overall survival analysis. Overall survival (OS) comparison between antiviral and non-antiviral groups **(A)**, hepatitis B virus (HBV) reactivation and no HBV reactivation groups **(B)** using Kaplan-Meier method. OS was calculated from the beginning of HAIC to death resulting from hepatocellular carcinoma (HCC). The difference was significant if p<0.05 by log-rank test.

As shown in **Figure 2A**, patients in antiviral group had a better OS compared with patients in non-antiviral group (p<0.05). Median OS was 16.46 months (95% CI, 15.25–17.67 months) in antiviral group and 10.68 months (95% CI, 7.44–13.92 months) in non-antiviral group. However, there was no significant difference in OS between patients developed HBV reactivation (median 11.34 months, 95% CI 7.79–14.88 months) and those without HBV reactivation (median 16.13 months, 95% CI 12.84–19.43 months) (p=0.135), although the former tended to have shorter survival time than the latter (**Figure 2B**).

## Discussion

Locoregional and systematic treatment such as TACE, hepatectomy, RFA, radiotherapy, systemic chemotherapy and immunotherapy could cause HBV reactivation; however, few studies focused on the effect of HAIC on HBV reactivation. In order to explore the occurrence of HBV reactivation in HCC patients treated with HAIC and the role of antiviral prophylaxis, we carried out this study.

The incidence of HBV reactivation in HBsAg positive HCC patients received no antiviral prophylaxis at baseline was reported approximate 14.5%–40.5% after TACE (13, 22), 20.7%–27.8% after hepatectomy (14, 15), 7.6% after RFA (14), 36.3% after systematic chemotherapy (combination cisplatin, interferon, doxorubicin and fluorouracil, or single-agent doxorubicin) (9), and 3.6% after immune checkpoint inhibitors (10), respectively. In our study, the incidence of HBV reactivation was 27.3% (9/33) after HAIC in the non-antiviral group, which was located between the incidence of TACE (14.5%–40.5%) and systemic chemotherapy (36.3%). As HAIC is a kind of treatment modality with the characteristics of both systemic chemotherapy and local interventional therapy, the data were in accordance with expectations.

Although HBV reactivation can occur spontaneously, it usually occurs after chemotherapy or immunosuppressive therapy, in which situation, the extent of immunosuppression caused by treatment is closely related to the incidence and severity of HBV reactivation (25, 26). As previously reported, cytotoxic chemotherapy drugs may inhibit the immunity of the body and interfere with the host’s immune monitoring of HBV replication, in which process the body’s immunity is a critical factor in controlling virus replication. When receiving chemotherapy drugs, the function of lymphocytes could be suppressed and a variety of immune related pathways, including the production of viral inhibitory cytokines such as interferon gamma (IFN-γ) and tumor necrosis factor (TNF) might also be inhibited, followed by the increased replication of HBV accordingly (27, 28). In addition to systemic chemotherapy, locoregional treatments, especially TACE, can also cause HBV reactivation in HCC patients; however, the underlying mechanism remains unclear. Unlike systemic therapy, TACE has limited systemic effect on host immunity through chemotherapy agents, as chemotherapy drugs used in TACE need to be carried by microspheres, which will greatly limit the dose of chemotherapy drugs compared with systemic chemotherapy, although the commonly used doxorubicin/epirubicin has the potential to activate HBV (16). More likely, TACE exerts systemic effect through arterio-venous shunt or altering tumor microenvironment, thereby impairing host immune regulation (29). HAIC is a strategy of local treatment through hepatic artery with systemic chemotherapy dosage, which has achieved good clinical effect in advanced HCC (30). HAIC is characterized by use of the same large-dose chemotherapy drugs as systemic chemotherapy with high local concentration, as well as exerting negative impact on the local immune microenvironment that similar to TACE, which might explain why HAIC can bring about HBV reactivation to some extent.

Compared with non-antiviral group, the morbidity rate of HBV reactivation in antiviral group decreased significantly in the patients treated with TACE (from 17.5% to 1.5%) (13), hepatectomy (from 27.8% to 3.0%) (15), and RFA (from 7.6% to 0%) (14), respectively. Consistent with previous studies, we also revealed that non-antiviral prophylaxis was the only risk factor for HBV reactivation and antiviral therapy at baseline evidently reduced the incidence of HBV reactivation in patients receiving HAIC (from 27.3% to 11.7%). Later follow-up showed that about 40% of patients in the antiviral treatment group took antiviral drugs irregularly, which might result in a relative high occurrence rate of HBV reactivation.

Among the 25 patients who developed into HBV reactivation, the only statistically significant difference between the antivirus and non-antivirus groups was that the former received more cycles of HAIC (3.11 ± 1.69 vs 1.75 ± 1.18). In other words, antiviral therapy can delay, if not prevent, the reactivation of HBV in patients undergoing HAIC.

The expert consensus suggested that long-term adjuvant nucleoside analogs (NAs) antiviral treatment should be provided to HBV DNA positive HCC patients; while HBV DNA negative HCC patients undergoing TACE, systematic chemotherapy or radiation therapy, NAs should be administered before the anti-cancer therapy (31). In the present study, we found that HBV reactivation could occur in HBsAg positive patients regardless of whether the baseline HBV DNA was measurable or not (no significant difference). Therefore, NAs antiviral treatment should also be administered before HAIC no matter HBV DNA level if only the HBsAg is positive. The vast majority of patients enrolled in our research took entecavir, and only a small proportion of patients took other NAs such as lamivudine, adefovir and telbivudine, thus no obvious differences in the prevention of HBV reactivation between several antiviral drugs were observed. Current guidelines concerning antiviral treatment for CHB patients recommend entecavir or tenofovir as the first-line treatment and lamivudine, adefovir or telbivudine as the second-line treatment (32, 33). However, due to the relatively short survival time of advanced HCC patients receiving HAIC, there should be little opportunity of developing into drug resistance, and the selection of antivirus agents may differ from that of CHB patients. For patients with longer expected survival time, antiviral therapy should be selected according to the practical guidelines for CHB treatment due to the possible drug resistance over time.

According to previous report, the typical clinical process of HBV reactivation generally includes three phases: Phase 1 refers to the start-up of HBV reactivation with a sudden increase in HBV replication after undergoing interventions with immunosuppressive effect. Phase 2 means that when the immunosuppression is weakened or just removed, the patient’s hepatocytes appear obvious injury and inflammation, and serum ALT level increases significantly, sometimes accompanied by jaundice. Phase 3 is the convalescent period, the damage of hepatocytes is partially alleviated, and the copy number of HBV DNA falls to the baseline level or below. Clinically, not all HBV reactivated patients will experience this typical process. In some patients, the serum HBV DNA level increase several folds, but the liver cells are not damaged obviously, with no change or only slight increase in serum ALT level (25, 34). In our investigation, seven of the 25 HBV reactivation patients developed hepatitis without jaundice, and the liver function of these seven patients ultimately recovered to normal status due to active antiviral and supportive therapy. The remaining 18 patients did not experience the typical three phases of HBV reactivation. It could be inferred from our study that only part of patients with HBV reactivation will develop hepatitis and HBV related hepatitis can be reversed through close monitoring and active treatment in HCC patients undergoing HAIC.

Previous studies have confirmed the efficacy of antivirus treatment on improving disease free survival (DFS) and OS of HBV related HCC patients receiving radical resection and TACE (35, 36). Nevertheless, evidence is still lacking regarding the influence of antivirus therapy on the prognosis of HCC patients underwent HAIC. Our results demonstrated that antiviral therapy can significantly prolong the OS of HCC patients compared with non-antiviral patients, which was consistent with those previously reported in surgery and TACE. As no significant difference in OS was observed in terms of whether or not HBV was reactivated, antiviral therapy might not improve survival just through preventing HBV reactivation. The specific mechanism is still not very clear. In theory, NAs therapy can promote virus clearance, inhibit the activity of hepatitis, reduce the chronic hepatic inflammation, and reverse liver fibrosis after treatments. It may also extend patients’ survival time by keeping liver function reserve and allowing patients to tolerate more cycles of anticancer treatment (37, 38).

The treatment information after HAIC was lacking, so the survival analysis was not accurate enough because the following treatment may also affect the prognosis, which was a limitation of the present study.

## Conclusion

In summary, based on our study, HAIC using mFOLFOX regimen can cause HBV reactivation in HBsAg-positive HCC patients, which needs close monitoring during and after HAIC. The use of NAs prophylactic antiviral therapy can effectively prevent or at least delay the occurrence of HBV reactivation. In addition, antiviral therapy can also prolong the overall survival of HCC patients receiving HAIC.

## Data Availability Statement

The original contributions presented in the study are included in the article/supplementary materials. Further inquiries can be directed to the corresponding authors.

## Ethics Statement

The studies involving human participants were reviewed and approved by Research Ethics Committee of Sun Yat-sen University. The patients/participants provided their written informed consent to participate in this study.

## Author Contributions

Conceptualization, all the authors. Data curation, SL, JL, NL, BZ, and MZ. Formal analysis, QX, HC, DC, and MH. Funding acquisition, MZ. Investigation, SL, JL, and NL. Methodology, SL, JL, NL, BZ, and MZ. Project administration, BZ and MZ. Resources, SL and MZ. Software, QX, HC, DC, and MH. Supervision, BZ and MZ. Validation, BZ and MZ. Visualization, SL, BZ and MZ. Writing—original draft, SL. Writing—review and editing, all the authors. All authors contributed to the article and approved the submitted version.

## Funding

This research was funded by 5010 Supporting Project for Clinical Medical Research of Sun Yat-Sen University.

## Conflict of Interest

The authors declare that the research was conducted in the absence of any commercial or financial relationships that could be construed as a potential conflict of interest.

## Abbreviations

AFP, alpha-fetoprotein; ALT, alanine transaminase; CHB, chronic hepatitis B; DFS, disease free survival; HAIC, hepatic artery infusion chemotherapy; HBsAg, hepatitis B surface antigen; HBV, hepatitis B virus; HCC, hepatocellular carcinoma; HIV, human immunodeficiency virus; HR, hazard ratio; NAs, nucleoside analogs; OR, odds ratio; OS, overall survival; RFA, radiofrequency ablation; TACE, transarterial chemoembolization.
